# Fermentation of Cauliflower and White Beans with *Lactobacillus plantarum* – Impact on Levels of Riboflavin, Folate, Vitamin B_12_, and Amino Acid Composition

**DOI:** 10.1007/s11130-020-00806-2

**Published:** 2020-03-06

**Authors:** H. O. Thompson, G. Önning, K. Holmgren, H. S. Strandler, M. Hultberg

**Affiliations:** 1 Department of Biosystems and Technology, Swedish University of Agricultural Sciences, Alnarp, Sweden; 2 Probi AB, Ideon Gamma 1, Lund, Sweden; 3 Biomedical Nutrition, Pure and Applied Biochemistry, Center for Applied Life Sciences, Lund University, Lund, Sweden; 4 Swedish Food Agency, Box 622, SE - 751 26 Uppsala, Sweden

**Keywords:** B-vitamins, *Brassica oleracea*, Lactic acid bacteria, Nutritional quality, *Phaseolus vulgaris*

## Abstract

As diets change in response to ethical, environmental, and health concerns surrounding meat consumption, fermentation has potential to improve the taste and nutritional qualities of plant-based foods. In this study, cauliflower, white beans, and a 50:50 cauliflower-white bean mixture were fermented using different strains of *Lactobacillus plantarum*. In all treatments containing cauliflower, the pH was reduced to <4 after 18 h, while treatments containing only white beans had an average pH of 4.8 after 18 h. Following fermentation, the riboflavin, folate, and vitamin B_12_ content of the cauliflower-white bean mixture was measured, and compared against that of an unfermented control. The riboflavin and folate content of the mixture increased significantly after fermentation. Relative to control samples, riboflavin increased by 76–113%, to 91.6 ± 0.6 μg/100 g fresh weight, and folate increased by 32–60%, to 58.8 ± 2.0 μg/100 g fresh weight. For one bacterial strain, *L. plantarum* 299, a significant 66% increase in vitamin B_12_ was observed, although the final amount (0.048 ± 0.013 μg/100 g fresh weight) was only a small fraction of recommended daily intake. Measurements of amino acid composition in the mixture revealed small increases in alanine, glycine, histidine, isoleucine, leucine, and valine in the fermented sample compared to the unfermented control.

## Introduction

Recent research has highlighted good potential of a change in diet in helping to resolve global challenges such as climate change, biodiversity loss, and food insecurity [1]. Studies exploring future sustainable food systems in the Nordic countries suggest decreasing consumption of meat by 80–90% and increasing consumption of vegetables. Legumes, with their high protein content, are of special importance in this concern due to their benefits for agricultural cropping systems via biological nitrogen-fixation [2]. Thus, there is a need for developing new plant-based products including legumes.

Fermentation has been used since ancient times for food preservation, while also having an impact on organoleptic characteristics. Several traditional Asian fermented bean products have now become popular in the West, including tofu, tempeh, and miso. Additional driving forces in developing fermented vegetable products are the growing interest in locally produced food, and consumer interest in products with less chemical additives.

During fermentation with lactic acid bacteria (LAB), available carbohydrates are converted to organic acids, mainly lactic acid and acetic acid, depending on the species used. For vegetables, a decrease in pH to around 4 has been reported to ensure a stable product [3]. The dominant species in spontaneous lactic acid fermentation of vegetables is *Lactobacillus plantarum* [4]. A benefit of using well-known lactic acid bacteria, such as *L. plantarum,* for fermentation is that they are included in the Qualified Presumption of Safety (QPS) list, which authorizes their use in the food and feed chain within the European Union.

The aim in lactic acid fermentation is generally to preserve the food by excluding growth of spoilage microorganisms. However, lactic acid fermentation is a strain-dependent and complex process with a broad impact on the nutritional value of the food [5]. An increase in the content of important nutrients, including the B-vitamins, after fermentation of plant-based products has been reported [6]. Apart from the direct effect on the food due to the bacterial metabolism, certain strains of LAB are also associated with probiotic properties. The *L. plantarum* strains investigated in the present study have been shown to have different health effects in humans, for example improved symptoms in people with irritable bowel syndrome [7], protection against lumbar spine bone loss in postmenopausal women [8], and increased iron absorption from foods [9].

The growth and capability for efficient fermentation of LAB are affected by several factors, such as composition of the substrate, strain-specific variations, and the fermentation procedure. In the present study fermentation of vegetables, cauliflower, white beans, and a mixture (50:50) of cauliflower and white beans, was studied. Four strains of *L. plantarum* were used and the effects of fermentation on levels of important nutritional parameters such as amino acid composition and riboflavin, folate, and vitamin B_12_ content were studied. Three of the investigated strains are available commercially as food supplements and as chilled plant-based food products and have been used to ferment cereals, berries and fruit [10–12]. The ability of the included strains to ferment vegetables and produce riboflavin, folate and vitamin B_12_ have not been investigated before.

## Material and Methods

### Bacterial Cultures

Four different strains of *L. plantarum* (strain 299v, strain Lp900, strain 299, strain Heal19) were provided by the company Probi AB, Sweden (https://probi.com/) and are described in Table 1. For production of the *inoculum* used for fermentation, the strains were cultivated as static culture in MRS broth (BDH Chemicals, UK) at 35 °C for 16 h. After this, the cells were harvested by centrifugation (Eppendorf MiniSpin, 10,000 g for 3 min) and washed twice with 0.85% NaCl solution. The control treatment was prepared with sterile MRS broth and a similar washing procedure. The bacterial suspensions, diluted in 0.85% NaCl and with an OD_620_ of 0.8 (corresponding to 7–8 log CFU/ml), and a control suspension (0.85% NaCl only) were added in a concentration of 1% (*w*/w) to the vegetable mixtures.

**Table 1:** Strains of *Lactobacillus plantarum* used in this study

Strain	Origin	DSM number^1^
*L. plantarum* strain 299v	Human gastrointestinal (GI) tract	9843
*L. plantarum* strain Lp900	Ogi, red sorghum, Nigeria	–
*L. plantarum* strain 299	Human GI tract	6595
*L. plantarum* strain Heal19	Human GI tract	15,313

The strains deposited at DSM are available commercially as food supplements and as chilled plant-based food products

^1^German Collection of Microorganisms and Cell Cultures

### Experimental Set-up

Raw cauliflower (*Brassica oleracea var. botrytis*) mixed in food processor, cooked and mixed white beans (*Phaseolus vulgaris* L.), and a mixture of consisting of a 50:50 ratio (*w*/w) of raw cauliflower and cooked white beans (cauliflower-white bean) were weighed out into plastic containers. Each portion weighed 100 g ± 1 g and was combined with 2 g of sea salt.

Suspension of *L. plantarum* was added to each container. The mixture was thoroughly stirred again following addition of bacteria or control suspension. The pH of each sample was recorded and the control samples were directly frozen at −80 °C. Containers with bacterial culture were incubated at 30 °C for 44 h. The pH of fermented samples was measured after 18 and 44 h.

After 44 h, the treatments were tasted and the cauliflower-white bean mixture was chosen for further analysis. Samples for determination of riboflavin (vitamin B_2_), folate, vitamin B_12_, total protein, and amino acid composition in this treatment were prepared and frozen at −80 °C. All samples were analyzed for total protein and vitamin content, while analyses of amino acid composition were performed on the control samples of cauliflower-white bean mixture and the samples fermented with *L. plantarum* 299.

### Analysis

#### Determination of Riboflavin, Total Amount of Folate and Vitamin B_12_

Determination of riboflavin was performed according to European Standard EN14152, as described by Jakobsen [13]. Determination of the total amount of folate was performed according to European Standard EN1413, as described by DeVries *et al*. [14], except for use of protease in the extraction procedure. Extraction of vitamin B_12_ was performed according to method AOAC 952.20, as described by Ball [15].

#### Total Protein and Amino Acid Composition

Total amount of protein in the samples was determined by the Dumas method [16] and applying a conversion factor of 6.25 for total nitrogen. Concentrations of the amino acids were determined according to the method of Llames and Fontaine [17].

### Statistics

The experiments were set up with three replicates in each treatment and repeated once. The data obtained were analyzed statistically using Minitab 17 for Windows. One-way Anova followed by Tukey’s multiple comparison test was employed to test for effects of treatments and the significance level was set to *P ≤* 0.05.

## Results and Discussion

**Fig. 1:**
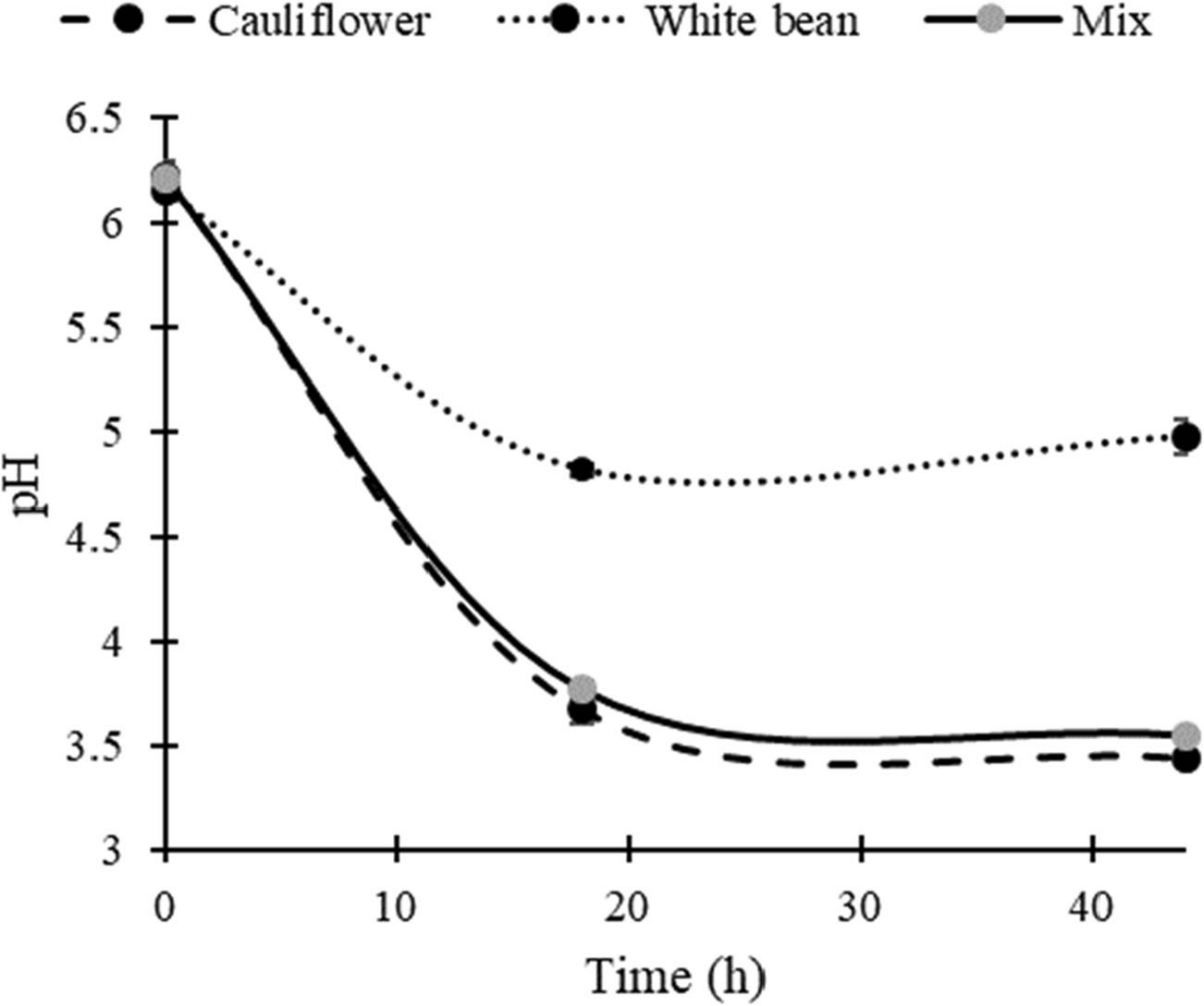
Changes in pH during fermentation with *Lactobacillus plantarum* of homogenized cauliflower, white bean, and a mix (50:50) of cauliflower and white bean

The different strains of *L. plantarum* behaved similarly with regard to the effect of pH on the different treatments. No significant difference was observed between the strains within each reading (18 and 44 h). Data from all strains were therefore pooled for each time point for analysis of the effect of fermentation on pH (Fig. 1). Cauliflower and white bean had a similar initial pH of approximately 6.20. However, after 18 h of fermentation with *L. plantarum* strains, the pH was significantly lower in the cauliflower and the cauliflower-white bean mixture treatments (3.66 ± 0.05 and 3.75 ± 0.04, respectively, mean ± SD) than in the treatment with white bean only (4.82 ± 0.02). After 44 h of fermentation, a slight but significant increase in pH was observed in the white bean treatment, to 4.96 ± 0.01. In the treatments with cauliflower and cauliflower/white bean mixture the opposite pattern was observed, with a slight but significant decrease in pH to 3.44 ± 0.1 and 3.52 ± 0.07, respectively.

Thus, fermentation was more efficient in the treatments including cauliflower adding benefits such as increased shelf life due to the low pH. Cauliflower is reported to contain approximately 4.2% carbohydrates, with a high concentration of monosaccharides [18]. Legumes, on the other hand, are well-known for containing high amounts of complex oligosaccharides, a component of dietary fiber that is less available for microbial degradation [19]. Thus, the easily available carbohydrates provided by cauliflower most probably sustained microbial growth, followed by a decrease in pH due to production of organic acids, to a greater extent than in the white bean treatment.

The mixture of cauliflower and white bean was chosen for further studies on the content of total protein, riboflavin, folate, and vitamin B_12_. The taste was dominated by a sour and salty flavour, similar to traditional fermented cabbage (sauerkraut), but with a deeper, underlying umami taste that brought a mild cheese-like quality. The taste was unusual, but not unpleasant, though more comprehensive taste analysis and consumer research would be required to determine the marketability of the product.

From a nutritional perspective the benefit of including white beans was clearly apparent, as the total amount of protein ranged from 21.1 to 23.2% of dry weight in the different bacterial treatments. Inconsistent results have been reported regarding the effect of fermentation on total protein amount [20]. Based on work with cereals, it has been suggested that the total amount of protein is generally not changed during lactic acid fermentation, although an increase can be observed in certain cases. If an increase is observed, it can often be related to a decrease in carbon ratio in the total mass due to bacterial metabolization of carbohydrates [21]. In the present study, no significant differences were observed in organic carbon content or in total nitrogen in relation to the control or between any of the treatments. Based on these results, no effect on the total protein amount was observed.

Riboflavin is important for the function of several enzymes involved in energy metabolism. It is naturally present in several different foods, including plants, and main sources of riboflavin intake are milk and dairy products, followed by cereals and meat. It is a water-soluble vitamin that is not stored in the body, and the daily dietary reference value has been set to 1.6 mg for adults [22]. Despite its presence in a wide variety of foods, riboflavin deficiency may occur. In this study, fermentation with *L. plantarum* increased the concentration of riboflavin significantly compared to the control in all treatments (Table 2). Significant differences in riboflavin concentration related to the different strains were observed. The highest value was observed after fermentation with *L. plantarum* Lp900, which gave an increase of 113% compared to the initial value, to 91.6 ± 0.6 μg/100 g fresh weight. The smallest increase, 76% of the initial value, was observed in the treatment with *L. plantarum* 299v. A similar increase in riboflavin content has been observed by Capozzi *et al*. [23] on fermenting wheat with *L. plantarum* for production of bread and pasta. However, in their study the strains used were selected for over-production of riboflavin, while such selection was not applied in the present study. Our results suggest that fermentation with *L. plantarum* can be used to increase the concentration of riboflavin in plant-based foods. However, it should be pointed out that the levels of riboflavin in the final fermented product were still generally low, at the level of μg/100 g product, compared to the recommended daily intake of 1.6 mg.

**Table 2:** Concentration (μg/100 g fresh weight) of riboflavin, folate, and vitamin B_12_ before (control) and after lactic acid fermentation of a mixture of cauliflower and white beans at 30 °C for 44 h using four different strains of *Lactobacillus plantarum*

Treatment	Riboflavin	Folate	Vitamin B_12_
Control	42.83 ± 1.20a*	36.84 ± 0.81a	0.029 ± 0.002a
*L. plantarum* strain 299v	75.64 ± 0.82b	58.82 ± 1.98c	0.033 ± 0.004ab
*L. plantarum* strain Lp900	91.60 ± 0.56c	55.88 ± 0.98c	0.034 ± 0.011ab
*L. plantarum* strain 299	76.36 ± 9.21b	48.74 ± 3.98b	0.048 ± 0.013b
*L. plantarum* strain Heal19	85.07 ± 2.14bc	53.55 ± 1.28bc	0.034 ± 0.004ab

Values shown are mean ± standard deviation

*Different letters within columns indicate significant differences (*p* ≤ 0.05; Anova followed by Tukey’s test)

For folate, a similar pattern as for riboflavin was observed, with a significant increase in all fermented samples and variations between strains. Like riboflavin, folate is synthesized by both plants and microorganisms, with main dietary sources being leafy green vegetables, dairy products, and cereal products. This vitamin, including several related compounds play a key role in ensuring essential functions of cell metabolism, such as DNA synthesis. However, the bioavailability of natural food folates varies and these compounds are easily degraded. Thus, folate deficiency is a general concern, and a strategy based on fortification of selected foods has been adopted in some countries. In this study, the highest concentration of folate was observed after fermentation with *L. plantarum* 299v, which showed an increase of 60% compared to the initial value, to a total concentration of 58.8 ± 2.0 μg/100 g fresh weight. The smallest increase, 32% of the initial value, was observed in the treatment with *L. plantarum* 299. Considering the average recommended intake of 250 μg dietary folate equivalents/day [24], the fermented vegetable mixture is of interest. The ability of microorganisms to produce folate is strain-specific, and a decrease in folate concentration in fermented products due to microbial consumption has been reported [25]. It should be pointed out that a significant increase in folate concentration was observed for all four strains of *L. plantarum* included in the present study, and that the genes for folate biosynthesis have been identified in this species [26]. Thus, fermentation of vegetables with *L. plantarum* might be considered as a general measure to increase folate concentration.

Vitamin B_12_ has a function as an important co-factor in several enzymes in procaryotes, protists, and animals, while B_12_-dependent enzymes have not been found in plants and fungi. Production of vitamin B_12_ has been shown to be limited to a few species of bacteria and archaea [27], and ensuring intake of adequate levels of this vitamin is a high concern with plant-based diets. In recent years, two strains of *L. plantarum* that produce vitamin B_12_ have been isolated [28]. In the present study, the increase in vitamin B_12_ in the fermentation treatments was less pronounced than that seen for riboflavin and folate. A significant increase in B_12_ content was observed after fermentation with *L. plantarum* 299 only (Table 2). An increase of 66% (to 0.048 ± 0.013 μg/100 g fresh weight) compared to the initial value (0.029 ± 0.002) was observed in this treatment. Considering that intake of 4 μg vitamin B_12_*per* day has been set as adequate by EFSA [29], it is clear that the fermented products evaluated in the present study could only provide a very small fraction of the total requirement, despite the significant increase. For the two vitamin B_12_-producing strains of *L. plantarum* previously isolated, it has been demonstrated that increased production of vitamin B_12_ can be achieved by addition of a B_12_ precursor such as 5-aminolevulinate [28]. Thus, a future approach to increase the concentration of vitamin B_12_ in fermented vegetables could be to ensure high concentrations of precursors before fermentation. Also, as the presence of human inactive analogues, such as pseudovitamin B_12_, have been reported in LAB high-producing strains should be subjected to detailed chemical analysis including not only microbiological assay but also liquid chromatographic methods [30].

It should be pointed out that no cell lysing treatment was performed in the present study, apart from storage in the freezer (−80 °C), and that strains of *L. plantarum* have been demonstrated to have high stability when frozen [31]. Additionally, no difference in moisture content in any of the treatments compared to the control was observed after lyophilization (data not shown). Thus, the increased levels of vitamins observed in the present study did not represent an intracellular pool, and were not due to an increase in dry matter.

No distinguishable difference in taste could be detected in the mixture fermented with the different bacterial strains and, based on the significant increase in vitamin B_12_ level, the vegetable mixture fermented with strain *L. plantarum* 299 was chosen for analysis of amino acid composition. The results showed small increases in alanine, glycine, histidine, isoleucine, leucine, and valine in the fermented sample compared to the control (Table 3). Of these amino acids, histidine, isoleucine, leucine, and valine are essential in the human diet. Thus, fermentation with *L. plantarum* 299 can be considered to have slightly improved the protein quality of the vegetable mixture. In contrast, a recent study reported a decrease in protein quality after fermentation of pea proteins with *L. plantarum* [32]. In that study, high consumption of the sulfur-containing amino acids was observed and thus the authors recommend selection of species other than *L. plantarum* for fermentation. This discrepancy in results, despite working with the same species and a similar vegetable, reflects the strain-specific metabolism of *L. plantarum*, which has been suggested to be due to their diverse ecological niches [28]. It also highlights the need for working with several strains of the same species in order to draw sound conclusions on characteristics of the species.

**Table 3:** Amino acid (aa) composition (g/100 g protein, dry weight basis) of a mixture of cauliflower and white bean before (control) and after fermentation with *Lactobacillus plantarum* strain 299

Amino acid	Control	Fermented sample
Alanine	1.11 ± 0.01a*	1.13 ± 0.01b
Arginine	1.42 ± 0.04a	1.42 ± 0.05a
Aspartic acid	2.92 ± 0.04a	2.97 ± 0.01a
Cysteine	0.23 ± 0.01a	0.24 ± 0.01a
Glutamic acid	3.60 ± 0.04a	3.63 ± 0.03a
Glycine	0.97 ± 0.01a	1.03 ± 0.01b
Histidine	0.67 ± 0.01a	0.70 ± 0.01b
Isoleucine	1.09 ± 0.00a	1.13 ± 0.01b
Leucine	2.02 ± 0.01a	2.08 ± 0.02b
Lysine	1.80 ± 0.01a	1.84 ± 0.05a
Methionine	0.25 ± 0.01a	0.23 ± 0.03a
Phenylalanine	1.40 ± 0.04a	1.46 ± 0.03a
Proline	0.93 ± 0.07a	1.00 ± 0.03a
Serine	1.52 ± 0.02a	1.48 ± 0.02a
Threonine	1.09 ± 0.01a	1.10 ± 0.01a
Tyrosine	0.85 ± 0.02a	0.87 ± 0.01a
Valine	1.32 ± 0.02a	1.36 ± 0.01b
Ʃaa	23.21 ± 0.14a	23.68 ± 0.05b

*Values within rows followed by different letters are significantly different (*p* < 0.05)

## Conclusions

Lactic acid fermentation is of importance for food preservation, while also having impact on taste and nutritional composition. In the present study three different vegetable substrates (cauliflower, white bean, and cauliflower-white bean mixture) were fermented using four different strains of *L. plantarum*. All strains had a similar impact on pH of the different substrates, and fermentation was more efficient in the treatments including cauliflower. Due to the efficient fermentation, with a final pH below 4, the pleasant taste and inclusion of legumes the impact of fermentation on riboflavin, folate, and vitamin B_12_ concentrations and on protein quality was studied in the cauliflower-white bean mixture. All strains of *L. plantarum* significantly increased the content of both folate and riboflavin compared to an unfermented control. Fermentation also had an impact on the content of vitamin B_12_, with fermentation with one of the bacterial strains (*L. plantarum* 299) resulting in a significant increase in vitamin B_12_ content. In the treatment involving fermentation of a cauliflower-white bean mixture with *L. plantarum* 299, amino acid composition was analyzed. The results revealed small increases in the concentrations of alanine, glycine, histidine, isoleucine, leucine, and valine in the fermented sample compared to the unfermented control.

Thus a slight improvement in nutritional quality was obtained after fermentation, although it should be pointed out that the quantity of different vitamins produced during fermentation, particularly of riboflavin and vitamin B_12_, was low relative to the recommended intake.
